# Computational Identification of Triphala-Derived Sterol Compounds as Putative Agonists of the Human Takeda G Protein-Coupled Receptor (TGR5)

**DOI:** 10.3390/ijms27073130

**Published:** 2026-03-30

**Authors:** Yathindra Maruthi Prasad, Sneha Ramaiah Gowda, Nandita Shantamurthy, Allwin Ebinesar Jacob Samuel Sehar, Sirajunnisa Abdul Razack, Somdet Srichairatanakool, Yuvaraj Ravikumar

**Affiliations:** 1 Department of Biotechnology, Acharya Institute of Technology, Bangalore 560107, India; 2 Department of Biochemistry, Faculty of Medicine, Chiang Mai University, Chiang Mai 50200, Thailand

**Keywords:** Triphala, TGR5 agonist, anti-inflammatory, molecular docking, molecular simulations and dynamics

## Abstract

The presence of an unbalanced gut microbiome and the dysregulation of bile acid signalling are considered pivotal causes of various inflammation-based diseases. The Takeda G protein-coupled receptor (TGR5), TGR5 is a bile acid-responsive receptor that modulates inflammatory signalling pathways, making it an enticing molecular target for the discovery of novel anti-inflammatory agents. Herein, a comprehensive in silico approach was employed to identify potential TGR5 agonists from sterol-rich phytocompounds present in Triphala, a traditional polyherbal formulation. Using in silico computational methods, such as molecular docking and molecular dynamics simulations (MDS), we screened the putative agonistic potential of 10 phytocompounds obtained from *Terminalia chebula*, *Terminalia bellirica*, and *Phyllanthus emblica* against the crystal structure of human TGR5 (PDB ID: 7XTQ). Based on binding energy and molecular interactions, ergosterol (−12.34 ± 0.17 kcal/mol) and stigmasterol (−10.35 ± 0.04 kcal/mol) were predicted to be the top and best compounds. Furthermore, the stability of these two compounds in the docked complex was analysed using MDS for 200 ns. The mean Cα RMSD values were 0.22 ± 0.02 nm for both ergosterol- and stigmasterol-bound complexes, compared to 0.21 ± 0.02 nm for the unbound apo protein. Further, the molecular mechanics/Poisson–Boltzmann surface area (MMPBSA) analysis revealed that ergosterol exhibited binding free energy (−139.868 ± 12.318 kJ/mol) comparable to that of the co-crystallised ligand R399 −93.424 ± 8.919 kJ/mol. In silico ADMET predictions indicated acceptable drug-like properties and low toxicity for both compounds. Collectively, these computational findings suggest that ergosterol is a promising putative TGR5 agonist, warranting further experimental validation of its potential role in modulating inflammation-related pathways.

## 1. Introduction 

The human gut microbiome can be regarded as a substantial organ responsible for maintaining health and preventing a wide range of diseases [1]. Specific systemic processes, such as nutrient metabolism, gut immunity, and tumour development, are strongly influenced by the few million microbes residing in the gastrointestinal mucosa [2]. Like other vital organs, the gut microbiome’s legitimate function depends on its composition, which includes bacteria belonging to the phyla Actinobacteria, Bacteroidetes, and Firmicutes, and, to a lesser extent, Proteobacteria [3]. Massive alterations in the proportions among these phyla lead to a disease-causing imbalance, commonly referred to as dysbiosis [4]. Mounting evidence suggests dysbiosis can lead to the development of diseases such as inflammatory bowel disease, Crohn’s disease, ulcerative colitis, metabolic disorders, neurological disorders, autoimmune disorders and colon cancer [5]. One of the primary reasons underlying the dysbiosis is the use of non-steroidal anti-inflammatory drugs [6]. In addition, proton pump inhibitors can disturb the gut microbiome, leading to an overgrowth of Enterobacteriaceae and Bacteroidaceae [7]. Such an imbalance contributes to intestinal homeostasis and inflammation [8]. To abrogate the induced inflammation, it is essential to understand the molecular mechanisms that both drive and attenuate it.

**Figure 1:**
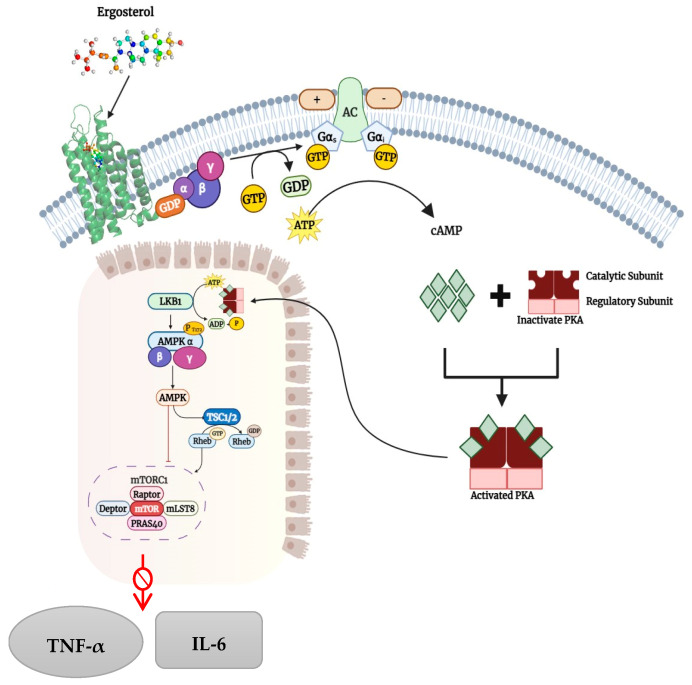
Proposed mechanism of TGR5-mediated anti-inflammatory signalling. TGR5 activation by known agonists, such as deoxycholic acid, induces Gαs-mediated stimulation of adenylate cyclase, leading to higher intracellular cAMP levels. Enhanced cAMP activates protein kinase A (PKA), which may modulate downstream signalling pathways, including AMPK activation and the suppression of mTORC1 activity, thereby decreasing the production of pro-inflammatory cytokines such as TNF-α and IL-6. In the present study, ergosterol was computationally predicted to bind to the TGR5 binding pocket. The downstream signalling pathway, as shown here, represents a hypothetical mechanism based on established TGR5 signalling. Experimental validation would be essential to confirm functional activation.

Intrinsically, Takeda G protein-coupled receptor (TGR5), also known as the G-protein-coupled bile-acid receptor (GPCR), is a membrane-bound receptor that exerts an anti-inflammatory protective mechanism [9]. Typically, TGR5 is activated upon binding to various bile acids. Among bile acids, lithocholic acid and deoxycholic acid exhibit promising binding affinity for TGR5 [10]. Upon activation with the agonist above, an increase in cyclic adenosine monophosphate (cAMP) levels was observed, which, in turn, downregulated inflammatory signals [11]. This bile acid signalling receptor has further demonstrated its immune-repressive effects on immune cells, particularly by reducing the secretion of tumour necrosis factor-alpha (TNF-α) and interleukin-6 (IL-6) by macrophages (Figure 1) [12]. Given its beneficial properties and natural tendency to exert anti-inflammatory effects, the discovery of novel agonists is of paramount importance. Although some semi-synthetic ligands, viz. 6α-ethyl-23(S)-methyl cholic acid and 3α,7β-dihydroxy-7α-methyl-5β-cholanoic acid, exhibited greater affinity for TGR5, the unknown side effects in a living system remain unclear [13]. Recently, the identification of novel compounds obtained from plant sources and the characterisation of their biological properties have spurred significant interest among researchers. This has become evident from the growing body of scientific evidence underscoring the diverse pharmacological potential of phytochemicals [14,15,16]. For instance, a diverse class of compounds, including flavonoids, alkaloids, phenolics, terpenoids, saponins, and tannins, has been shown to exhibit anti-inflammatory effects by reducing reactive oxygen species (ROS) levels, inhibiting NF-κB activation, suppressing cytokine release, and reducing the resulting inflammatory response [17,18]. Given the high curative effects of plant secondary metabolites, their scientific significance in combating dysbiosis-induced gut inflammation is paramount.

Earlier phytochemical studies on Triphala have confirmed the presence of a wide array of bioactive compounds, including ergosterol, stigmasterol, β-sitosterol, and other compounds, as well as tannins, flavonoids, and phenolic compounds [14]. Among these, β-sitosterol has been commonly reported to be present in *Terminalia chebula*, as has been identified through chromatographic and spectroscopic methods [15]. Along with their structural characterization, the identified sterol-like compounds present in Triphala have demonstrated certain antifungal, antimalarial and antiviral effects [18,19,20,21,22,23]. Furthermore, studies using in vivo experimental models of arthritis clearly show that these models exhibit anti-inflammatory and immunomodulatory properties. Earlier studies that have focused on the phytochemical investigation of Triphala have revealed that it contains a broad spectrum of phytoconstituents, including tannins, phenols, flavonoids, and phytosterols, such as ergosterol, stigmasterol, β-sitosterol, and others [19,24].

**Figure 2:**
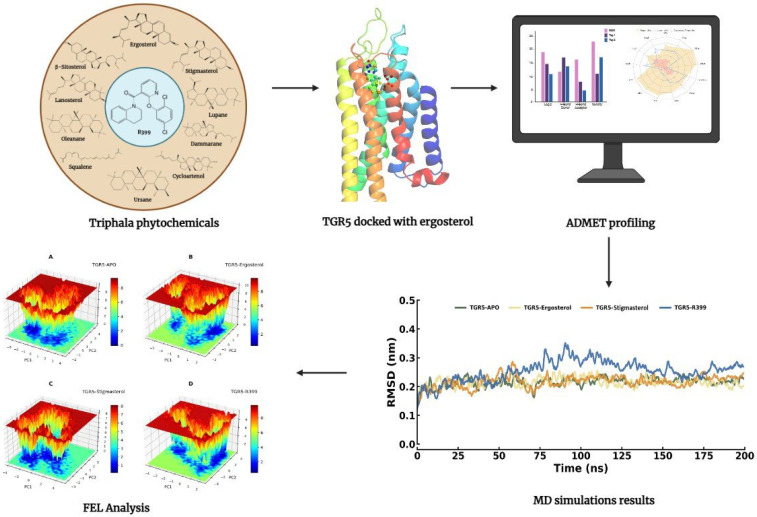
Graphical abstract summarising the computational workflow used to investigate the putative mechanism of action of TGR5 agonists.

The G protein-coupled bile acid receptor TGR5 is activated by conjugated and unconjugated bile acids, which possess a steroidal backbone. The phytosterols present in Triphala share a comparable polycyclic sterane core structure, suggesting potential structural compatibility with the TGR5 binding pocket. Although direct experimental evidence demonstrating TGR5 agonistic activity for Triphala-derived sterols is currently lacking, their structural features and reported pharmacological properties provide a rationale for exploring their interaction with this receptor. In recent years, computational drug discovery approaches have become valuable tools for identifying novel candidate ligands across various therapeutic areas, including cancer and hematological disorders [25,26]. In this context, we selected ergosterol, cycloartenol, β-sitosterol, lanosterol, dammarane, oleanane, lupane, ursane, and squalene for in silico screening against TGR5. Molecular docking was performed to evaluate binding compatibility, followed by molecular dynamics simulations and ADME/T analyses to assess complex stability and drug-likeness properties (Figure 2). These analyses were conducted to identify potential candidate ligands that warrant further experimental investigation.

## 2. Results

### 2.1. Molecular Docking of Selected Compounds Against TGR5

**Figure 3:**
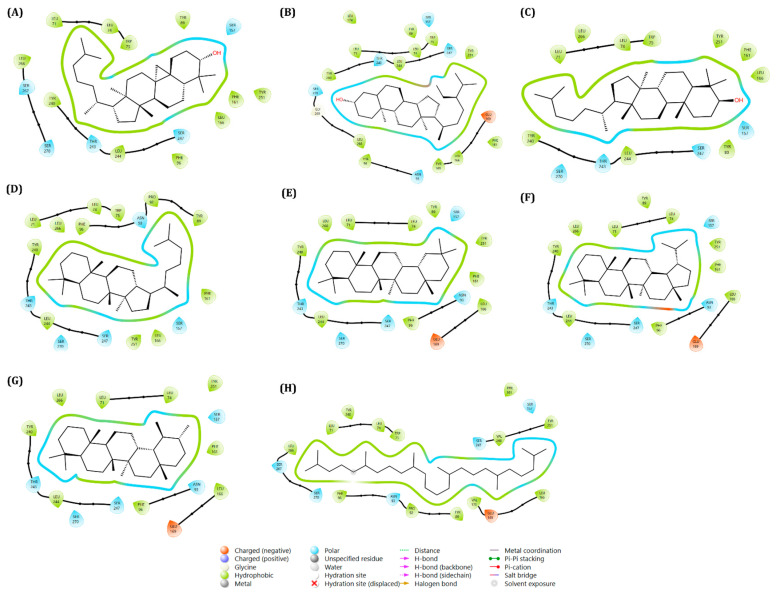
Two-dimensional (2D) interaction diagrams of selected phytosterol and triterpenoid compounds docked within the orthosteric binding pocket of TGR5 (PDB ID: 7XTQ): (**A**) Cycloartenol; (**B**) β-sitosterol; (**C**) Lanosterol; (**D**) Dammarane; (**E**) Oleanane; (**F**) Lupane; (**G**) Ursane; and (**H**) Squalene. Ligands are represented as ball-and-stick models, while interacting amino acid residues are shown as labelled circles. Residue labels correspond to amino acids located within the active-site cavity of TGR5.

**Figure 4:**
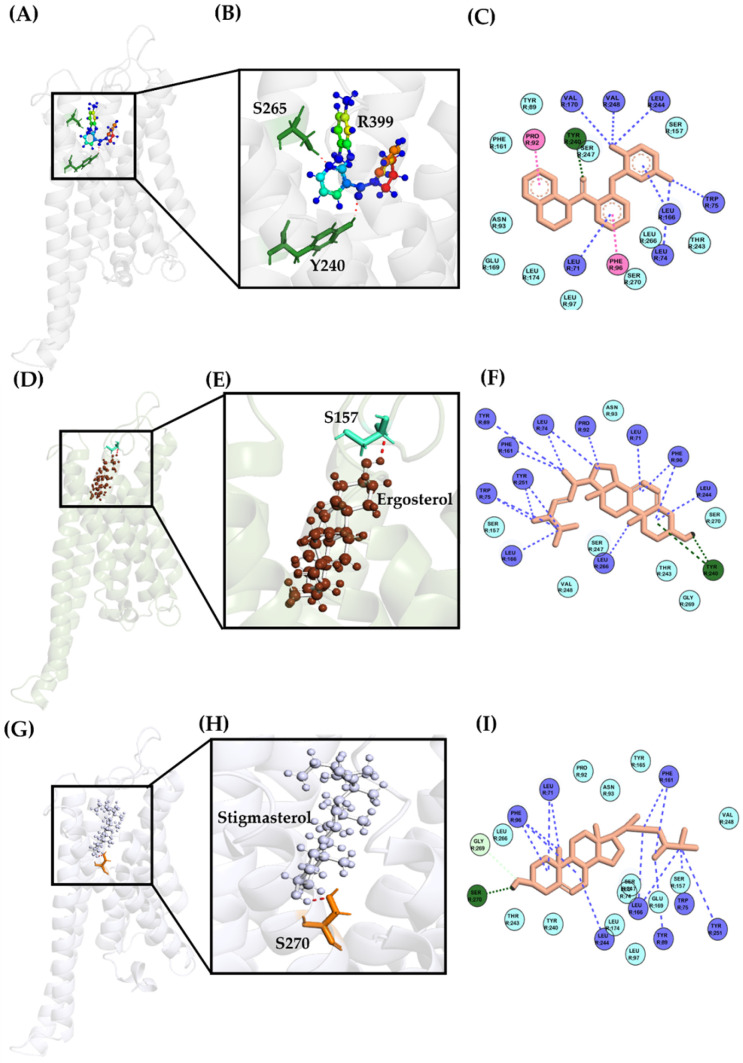
Structural and interaction analysis of TGR5 (PDB ID: 7XTQ) with co-crystallized and docked ligands. (**A**) Cartoon representation of the crystal structure of TGR5 (PDB ID: 7XTQ) showing the co-crystallized ligand R399 bound within the active site. (**B**) Three-dimensional (3D) close-up view of R399 within the binding pocket, highlighting key interacting residues (shown as sticks). (**C**) Two-dimensional (2D) interaction diagram of R399 with surrounding amino acid residues. (**D**) Cartoon representation of TGR5 showing the docking pose of ergosterol within the active site. (**E**) 3D close-up view of ergosterol bound to the receptor, with interacting residues displayed as sticks. (**F**) 2D interaction map illustrating hydrogen bonding and hydrophobic interactions between ergosterol and TGR5 residues. (**G**) Cartoon representation of TGR5 showing the docking pose of stigmasterol within the active site. (**H**) 3D close-up view of stigmasterol in the binding pocket with key interacting residues shown as sticks. (**I**) 2D interaction diagram of stigmasterol with TGR5 residues. In the 3D representations, interacting amino acid residues are shown as colored sticks, and interactions are depicted as dashed lines. In the 2D interaction maps, the ligand is shown as sticks, with hydrogen bonds represented by green dashed lines and hydrophobic interactions indicated by violet dashed lines.

To investigate the prospect of the binding ability of β-sitosterol, lanosterol, dammarane, oleanane, lupine, ursane, and squalene with the target protein TGR5, molecular docking was employed. The 2D and 3D structures of the docked compounds with the target protein TGR5 are shown in Figure 3 and Figure 4. TGR5 was selected as it played an essential role in the inflammatory response. Docking was performed using both the R399-bound (7XTQ) and INT-777-bound (7CXN) crystal structures to evaluate binding pocket conservation. The R399-bound TGR5 structure (PDB ID: 7XTQ) was selected as the primary docking template because it provided a high-resolution structure of the receptor in an active-like conformation with a well-defined hydrophobic binding cavity suitable for sterol-like compounds (Figure S1). Given that the screened phytosterols (ergosterol and stigmasterol) lacked the polar carboxylate moiety present in bile acid derivatives, we determined that the 7XTQ structure would be appropriate for this study. The interaction of the redocked R399 was similar to that of the co-crystal ones, particularly with regard to their relevant hydrogen bond interactions. Y240 was found to form a hydrogen bond interaction with R399, similar to that which was observed in the co-crystallised structure, with a docking score of −9.765 ± 0.005 kcal/mol. As shown in Figure S2, a close overlap between the crystallographic pose and the redocked pose indicates that the docking protocol could reliably reproduce the experimentally observed binding orientation, supporting the validity of the molecular docking procedure with an RMSD difference of 0.435 Å. Furthermore, as has been depicted in Table 1, ergosterol exhibited the strongest binding affinity among the tested compounds, with a binding energy of −12.34 ± 0.17 kcal/mol. This was followed by stigmasterol (−10.35 ± 0.04 kcal/mol), cycloartenol (−9.73 ± 0.52 kcal/mol), β-sitosterol (−9.27 ± 0.21 kcal/mol), lanosterol (−9.16 ± 0.03 kcal/mol), and dammarane (−8.83 ± 1.36 kcal/mol). In comparison, oleanane (−8.81 ± 0.70 kcal/mol), lupane (−6.35 ± 0.23 kcal/mol), ursane (−5.96 ± 0.34 kcal/mol), and squalene (−5.55 ± 0.55 kcal/mol) demonstrated weaker binding affinities. Notably, ergosterol, stigmasterol, and cycloartenol exhibited stronger binding capabilities than the positive control R399 (−9.77 ± 0.01 kcal/mol). After predicting the binding energy, the interaction arrangements of the compounds with TGR5 were examined. An analysis of the 2D and 3D diagrams for the docked complexes revealed that ergosterol, stigmasterol, cycloartenol, and β-sitosterol formed one hydrogen bond with the active site of TGR5. Furthermore, the best compounds were ranked not solely on the binding energy but also by considering the other parameters, such as the hydrogen bond and the total number of non-covalent interactions that existed within each docked complex. In light of these findings, we carefully assessed the total number and types of interactions formed for the R399 and all docked compounds. As shown in Figure 3 and Figure 4, we found that Y240 forms one hydrogen bond with ergosterol and one π-alkyl interaction, while L244, L266, L166, W75, Y251, F161, Y89, L74, P92, L71, and F96 formed π-alkyl and alkyl-type interactions with ergosterol. Likewise, in the stigmasterol-docked complex, S270 formed a hydrogen bond. Eight residues, namely F96, L71, F161, Y251, W75, Y89, L166 and L244, produced alkyl and π-alkyl interactions. Albeit alkyl and π-alkyl interactions are seen, no hydrogen bonds were formed with the docked complexes of lanosterol, dammarane, oleanane, lupane, ursane, and squalene. Notably, π-σ type interactions were most commonly observed in β-sitosterol, lanosterol, dammarane, oleanane, ursane, and squalene. F161 demonstrated the π-σ interaction with the ligands mentioned above, while an additional π-σ interaction was seen in β-sitosterol and ursane due to the aid of another aromatic amino acid, F96. Other stabilising interactions for all docked compounds are depicted as 2D representations in Figure 3. Considering that AutoDock 4.0 scoring functions have an estimated standard error of approximately 2–3 kcal/mol, small differences in binding energies should be interpreted cautiously. Therefore, ligand ranking was not based solely on docking scores but also on binding pose consistency and interaction patterns within the active site. As observed, interaction analysis showed that ergosterol formed one hydrogen bond with Y240. Similarly, stigmasterol formed one hydrogen bond with S270. In contrast, lanosterol, dammarane, oleanane, lupane, ursane, and squalene did not form hydrogen bonds within the binding pocket, and their stabilization was primarily mediated through hydrophobic and π-type interactions. F161 frequently contributed to π-σ interactions, while F96 additionally supported β-sitosterol and ursane binding. Given the combined consideration of the docking score and the interaction profile, ergosterol and stigmasterol were selected for further ADME/T evaluation and molecular dynamics simulations. Docking results were used as an initial screening step, and subsequent MD simulations were performed to validate the stability of the protein–ligand complexes.

### 2.2. Prediction of Drug-Likeness

The properties of drug-likeness for the top-ranked ergosterol and stigmasterol docked compounds were assessed on the grounds of Lipinski’s rule of five. This prediction is crucial for determining whether a compound has the physicochemical properties required for oral bioavailability. According to Lipinski’s rule, a compound can be treated as a drug if it bears the following properties: molecular weight (MW) ≤ 500, H-bond acceptors ≤ 10, H-bond donors ≤ 10, LogP ≤ 5, and molar refractivity between the range of 40 and 130. In accordance, the determined ADME properties for ergosterol and stigmasterol are summarised in Table 2. Both ergosterol and stigmasterol have molecular weights of 396.34 and 412.37 g/mol, which are well within the indicated range. The number of H-bond donors and acceptors for both top-ranked compounds remains 1.0. The LogP values for ergosterol and stigmasterol were 5.44 and 6.57, respectively, indicating that ergosterol is more drug-like. Further evaluation of the Topological Polar Surface Area (TPSA) indicates that both ergosterol and stigmasterol have TPSA values of 20.23, which fall within the Lipinski rule of 5. Furthermore, ergosterol has 4 rotatable bonds, and stigmasterol has 5. Taken together, the predicted physicochemical properties indicate that both compounds meet the key parameters set by Lipinski’s rule, including polarities of −5.125 and −5.733, suggesting probable drug likeness for the prospective studies.

### 2.3. Prediction of ADMET Properties

In drug discovery, predicting the properties related to the absorption, distribution, metabolism, excretion, and toxicity of novel probable drugs is essential to avoid undesirable outcomes during clinical phases. Thus, predicting bioavailability and pharmacokinetic properties should be considered inevitable, and, accordingly, the ADME of ergosterol and stigmasterol was determined using the predictive model pkCSM. In absorption, the Caco-2 permeability of ergosterol and stigmasterol is consistent with sound absorption, with predicted values of 16.5 × 10^−6^ cm/s, indicating effective intestinal permeability. A similar high permeability of 16.3 × 10^−6^ cm/s was observed for stigmasterol. Likewise, good intestinal absorption was predicted for both compounds. Furthermore, the logBB values for ergosterol (5.82) and stigmasterol (5.89) indicated high brain permeability. Alternatively, the CNS permeabilities of ergosterol (0.0177) and stigmasterol (0.0223) indicated low CNS permeability, and their effects on the CNS were likely to be minimal. In terms of metabolism, CYP3A4 inhibition was crucial, as it could increase plasma concentrations and enhance efficacy. It is also noteworthy to clarify that CYP3A4 metabolises most JAK3 inhibitors. We found that both compounds are substrates for CYP3A4, which indicates that this liver enzyme likely metabolises them. They also do not inhibit CYP1A2, CYP2C9, CYP2C19, CYP2D6, or CYP3A4, indicating that they do not inhibit the interaction of other drugs that are substrates for these enzymes. Further evaluation of primary toxicity testing using in silico methods would be invaluable, thereby reducing the time and expense needed for future animal studies. The toxicity of ergosterol and stigmasterol was estimated using ProTox-III, which assesses vital parameters including mutagenicity, carcinogenicity, hepatotoxicity, cellular toxicity, immunotoxicity, and Tox21 stress-related pathways. As shown in Table 3, both compounds are predicted to be non-mutagenic and non-carcinogenic. It is also noteworthy to evaluate the potential of these compounds to interfere with nuclear receptor and stress-signalling pathways. This would remain crucial, as various receptors are primarily proteins that function as ligand-activated transcription factors and can also induce cellular stress responses. As predicted, both ergosterol and stigmasterol remained inactive, suggesting that the compounds do not substantially affect the nuclear and stress-related signalling pathways.

### 2.4. Molecular Dynamics Simulations Analysis

MDS is an essential method for understanding the interactions and motions of the ligand-docked protein complex. It achieves this by mimicking the relevant pathological and physiological conditions. To understand the results of this analysis, diverse parameters, including root mean square deviation (RMSD), root mean square fluctuation (RMSF), and radius of gyration (Rg), were used. Solvent-accessible surface area (SASA) and hydrogen bonds (H-bonds) can be harnessed. RMSD quantified the mean deviation of the docked complex over time intervals and was used to evaluate structural stability. RMSF was used to evaluate atomic fluctuations and thus assess flexibility. Rg was used to indicate the degree of compactness of the docked complex and to help understand any conformational changes. SASA was used to determine the molecules’ accessible surface area to the solvent, thereby helping us understand their molecular interactions with the surrounding environment. Furthermore, H-bond analysis is vital in MDS, as it provides information on ligand–protein interactions. Taken together, the investigation of these parameters enables deeper insight into the biological function of the docked complex.

#### 2.4.1. RMSD

**Figure 5:**
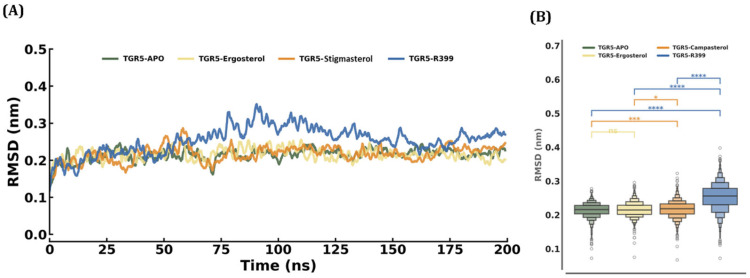
RMSD plot of each docked complex attained through MDS at 200 ns. (**A**) The blue colour indicates the RMSD of TGR5 (Apo). The yellow colour indicates the RMSD of TGR5 docked with the ergosterol. The orange colour indicates the RMSD of TGR5 docked to stigmasterol. The blue colour also indicates the RMSD of TGR5 docked to R399. (**B**) The box plot shows the RMSD distributions for each docked complex and the statistical significance relative to Apo, based on the RMSD values. *, ***, **** It denotes the significant value.

In this work, the stability of three docked complexes, TGR5-R399, TGR5–ergosterol, and TGR5–stigmasterol, as well as the apo form of TGR5, was simulated for 200 ns. The RMSD values for all the aforementioned species were computed and have been plotted in Figure 5. The determined RMSD values were grouped into 0.2–0.35 nm across all four complexes. The average RMSD values were recorded as follows: TGR5 Apo at 0.21 ± 0.02 nm, TGR5–ergosterol at 0.22 ± 0.02 nm, TGR5–stigmasterol at 0.22 ± 0.02 nm, and TGR5-399 at 0.25 ± 0.04 nm, respectively. A consistent stability of ~50 ns was observed in all simulation trajectories. The largest fluctuation was observed in the TGR5-R399 system, with a ~0.15 nm difference in RMSD values between 75 and 125 ns. The complexes with ergosterol (yellow) and stigmasterol (orange) maintained a highly stable conformation throughout the simulation (0–200 ns), except for a slight conformational change between 60 and 80 ns, which was also observed for the apo. Furthermore, the box plot shows the distribution of RMSD across all designated time intervals. As depicted in the figure, the RMSD distribution for ergosterol was ~0.20–0.23 nm. It closely resembled that of the apo system, with even the statistically insignificant values confirming that ergosterol binding to TGR5 did not change protein conformation. While stigmasterol and the control R399 exhibited a broader distribution of median RMSD, ~0.22 to 0.25 nm for stigmasterol and ~0.30 nm for R399, the statistically significant values further confirm moderate conformational fluctuation and R399’s greater conformational changes and stability when bound to the TGR5.

#### 2.4.2. RMSF

**Figure 6:**
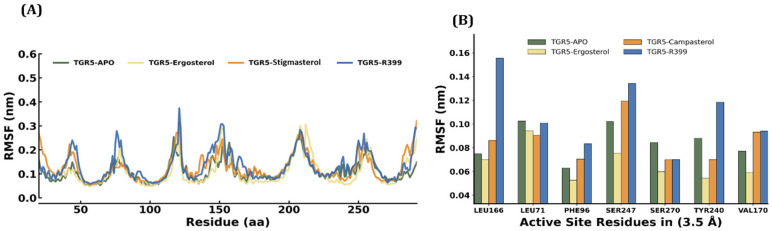
RMSF plot of each docked complex attained through MDS at 200 ns. (**A**) The blue colour indicates the RMSF of TGR5 (Apo). The yellow colour indicates the RMSF of TGR5 bound to ergosterol. The orange colour indicates the RMSF of TGR5 docked to stigmasterol. The blue colour also indicates the RMSF of TGR5 docked to R399. (**B**) The bar diagram shows the RMSF fluctuations of individual amino acids in the active-site region.

To investigate the flexibility of individual residues during MDS, the average RMSF values for TGR5-R399, TGR5–ergosterol, and TGR5–stigmasterol were measured relative to the Apo system. The mean RMSF values for the TGR5–Apo, TGR5–ergosterol, TGR5–stigmasterol, and TGR5-R399 docked complexes were 0.11 ± 0.04 nm. 0.11 ± 0.06, 0.13 ± 0.05, and 0.13 ± 0.06, respectively. As shown in Figure 6, a lower value was observed for the Apo form and the TGR5–ergosterol complex, indicating less flexible individual residues and more stable docked complexes. Albeit no substantial differences in the RMSF mean values were observed in the stigmasterol and R399 docked complexes, minimal variations in individual residues were observed. To further probe ligand-induced changes in the RMSF values of the active-site residues, we analysed the mean RMSF values of L166, L71, F96, S247, S270, Y240, and V170. Interestingly, we found that TGR5, upon binding to ergosterol, exhibited lower RMSF values for all the aforementioned residues than Apo and other docked complexes. Strikingly, a greater variation was observed in the TGR5-R399 complex than in Apo. In particular, L166 revealed an RMSF value ~0.08 nm higher, followed by S247 (~0.04 nm) and Y240 (~0.02 nm). Moderate variations were observed in the TGR5–stigmasterol complex, with lower RMSF values for S270 and Y240 than in the Apo state.

#### 2.4.3. Radius of Gyration and Solvent-Accessible Surface Area

**Figure 7:**
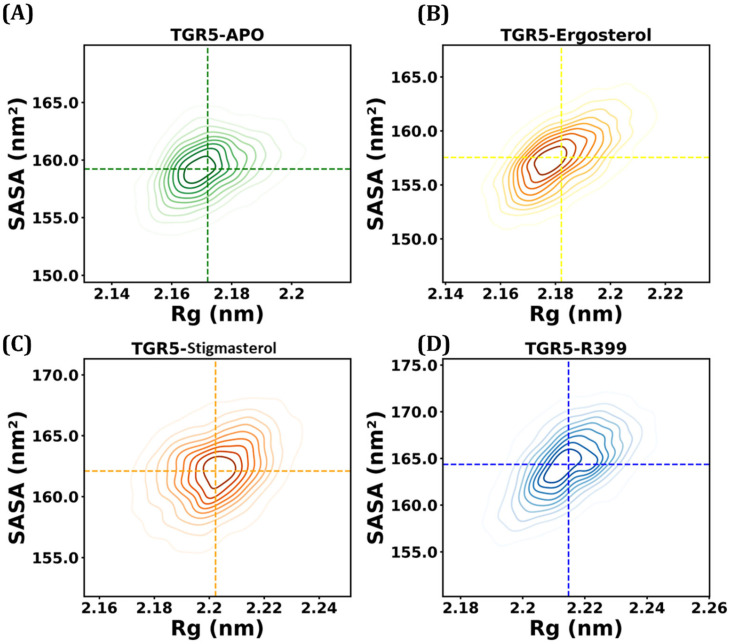
Kernel Density Estimation (KDE) plot analysis of Rg and SASA. (**A**) Green colour represents TGR5–Apo, (**B**) yellow colour represents TGR5–ergosterol, (**C**) orange colour represents TGR5–stigmasterol, and (**D**) blue colour represents TGR5-R399. The concentric circles represent the probability distribution as a function of intensity. It knows as center of axis.

MD trajectories for the TGR5-APO, TGR5-R399, TGR5–ergosterol, and TGR5–stigmasterol complexes were analysed using Rg and SAS. As Figure 7 shows, the average Rg values for all complexes were 2.17–2.21 nm over the entire time period. The mean Rg values for all the systems were as follows: TGR5-APO at 2.17 ± 0.01 nm, TGR5-R399 at 2.21 ± 0.01 nm, TGR5–ergosterol at 2.18 ± 0.01 nm, and TGR5–stigmasterol at 2.19 ± 0.01 nm, respectively. The ergosterol-bound complex (yellow) exhibited the lowest difference in Rg value (0.01 nm) when compared with APO and stigmasterol, while the R399-bound complex displayed a higher degree of fluctuation (0.03 nm). This would indicate that moderate disturbances in protein rigidity have occurred in the stigmasterol-bound samples when compared with the ergosterol-bound samples, and that R399 has exhibited the greatest change in protein rigidity, as indicated by the difference in the Rg value relative to Apo (0.15 nm). Furthermore, the detailed SASA prediction is advantageous for gauging the proteins’ structural stability upon solvent exposure throughout the 200 ns simulation. The estimated SASA values for the docked complexes and the apo form are presented in Figure 7. The estimated SASA values ranged from 159 to 164 nm^2^. The mean SASA values for TGR5-APO, TGR5-R399, TGR5–ergosterol, and TGR5–stigmasterol were 159.23 ± 2.18 nm, 164.37 ± 3.28 nm, 157.55 ± 2.63, and 162.10 ± 2.46 nm, respectively. These values clearly indicate that the ergosterol SASA values were closer to those of Apo, indicating that ligand binding did not induce any major conformational changes or significant protein unfolding. A slightly more exposed solvent conformation was observed for stigmasterol-1, with SASA values of ~168 nm^2^.

#### 2.4.4. Hydrogen Bond Interaction Analysis

**Figure 8:**
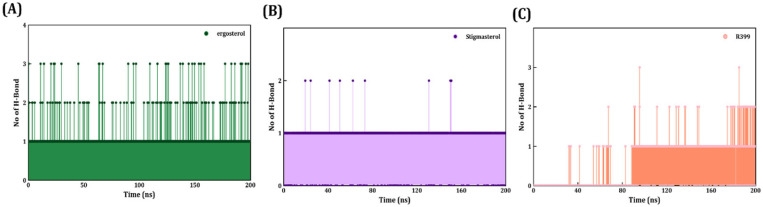
Analysis of H-bond interactions between each ligand bound to the protein through MDS. (**A**) The green line plot shows the inter-H-bond formed between TGR5 and ergosterol. (**B**) The purple line plot shows the inter-H-bond formed between TGR5 and stigmasterol. (**C**) The light-orange line plot shows the inter-H-bond formed between TGR5 and R399.

The evaluation of hydrogen bond interactions is crucial for discerning and illustrating ligand interactions with the protein using MDS analysis (Figure 8). Herein, we have determined the total number of hydrogen bonds formed between ergosterol, stigmasterol, and R399, as well as for the protein TGR5, over the entire 200 ns simulation. The average H-bond formed between TGR5 and ergosterol was four, and a maximum of five hydrogen bonds were seen forming over 200 ns, indicating that binding stability was influenced more by hydrogen bond interactions. In contrast, the average number of hydrogen bonds formed with stigmasterol appeared to be lower (two) in most frames, occasionally reaching three hydrogen bonds. This shows that hydrophobic interactions were more dominant than polar interactions during complex formation. In comparison with ergosterol and stigmasterol, R399 binding had moderate hydrogen bond interactions (three), but these interactions were more discontinuous. As depicted in Figure 8, it is clearly evident that no hydrogen bonds were noted continuously, even for single-hydrogen-bond formation. A comprehensive hydrogen bond study has revealed that ergosterol stabilisation via TGR5 was strongly mediated by hydrogen bonds, as evidenced by the 200 ns simulation.

#### 2.4.5. Principal Component Analysis

**Figure 9:**
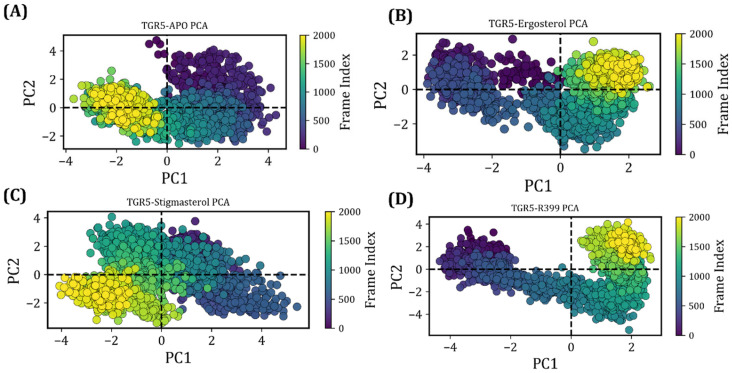
2D-projection of the principal component analysis shows the cluster conformation of each docked complex. (**A**) TGR5–Apo. (**B**) TGR5–ergosterol. (**C**) TGR5–stigmasterol. (**D**) TGR5-R399.

The molecular motions and conformational changes that occurred upon ligand binding were analysed using principal component analysis (PCA). The initial few eigenvectors (EVs) played a crucial role in the global motion of the protein molecule. Changes in the trajectory files generated a projection (i.e., eigenvector changes) by projecting the acquired trajectories onto two major components (PC1 and PC2). A matrix was constructed from the eigenvectors of the atoms in the molecule. This matrix illustrated how the protein’s atoms shifted. In view of this, the conformational diffusion of TGR5, along with its collective motions for Apo, ergosterol, and stigmasterol over 200 ns, was analysed. When compared with the ergosterol- and stigmasterol-bound complexes, distinct clusters were observed for ergosterol, specifically when separated by PC1, and these clusters tended to remain separated. Notably, clusters appeared much broader and were scattered across all quadrats with stigmasterol. Furthermore, a dominant, compact cluster with a narrow frame spread was observed in TGR5–ergosterol. An elongated and slightly broader distribution with too many clusters was shown across PC1 and PC2; this would suggest that the restricted motions and proteins tended to exist in the same conformation in the TGR5–ergosterol system. This shows that, upon binding to TGR5, ergosterol constrained TGR5’s conformational dynamics and stabilised conformations, whereas stigmasterol, which induced moderate conformational changes, limited stabilisation. With regard to R399, two distinct clusters were observed: one on the positive side of PC1 and one on the negative side. The clusters appeared much stronger, indicating that R399 binding led to a specific protein conformation (Figure 9).

#### 2.4.6. Free Energy Landscape Analysis

**Figure 10:**
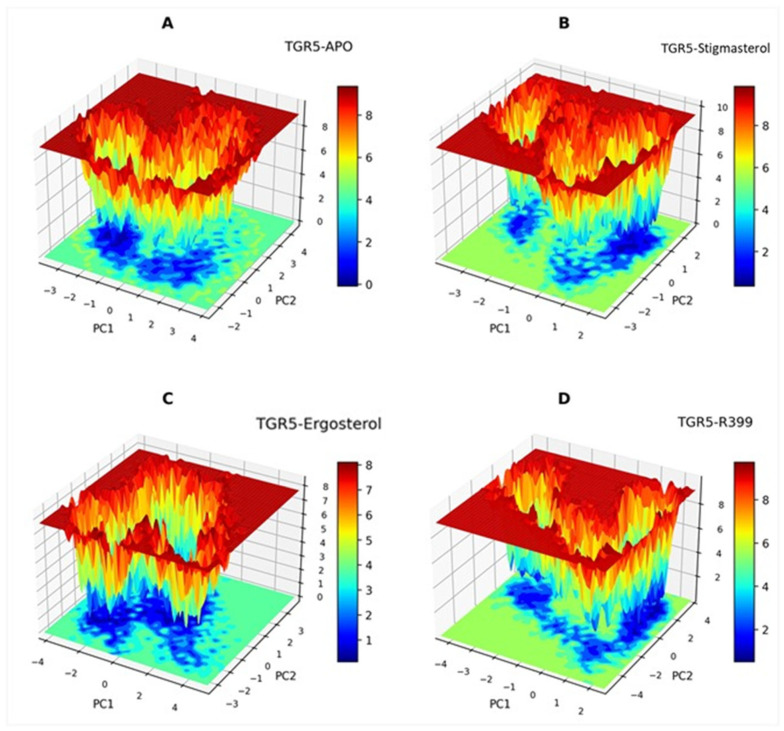
Three-dimensional representations of the free energy landscape for each ligand bound to the protein: (**A**) TGR5-Apo. (**B**) TGR5–stigmasterol. (**C**) TGR5–ergosterol. (**D**). TGR5-R399. The blue colour represents the conformational state with the minimum energy, in kJ/mol, and the red colour represents the state with the maximum energy, also in kJ/mol. The green colour represents the intermediate transition conformational states.

FEL is an invaluable approach used for detecting and measuring specific changes in the protein structure, such as protein aggregation, folding, and unfolding. The FEL results for the Apo, TGR5–ergosterol, TGR5–stigmasterol, and TGR5-R399 are presented in Figure 10. In TGR5-Apo, the landscape was broad and rough, comprising numerous shallow minima separated by low barriers. The complex TGR5–ergosterol exhibited fewer minima but deeper basins that were separated by substantial barriers. This demonstrates that entropy was low and that conformational selection occurred in one or two states, possibly upon binding to ergosterol. It is also noteworthy that a single, compact, low-energy basin was surrounded by high-energy contours, indicating the presence of energy barriers that could presumably prevent the formation of transition states. Furthermore, the absence of multiple low-energy minima would indicate that no competing conformations were thermodynamically accessible. These observations differed from those of TGR5–camposterol, where moderate roughness, often with too many basins, was observed. Additionally, elongated low-energy regions and the emergence of numerous metastable conformations indicated protein conformational switching due to reduced stability. Such presentations implied that stigmasterol binding led to multiple conformational states with limited stabilisation. Among the tested samples, R399 exhibited a firm, deep landscape, with one basin appearing very deep and sharp. Furthermore, several high barriers were observed, indicating that the conformational states were mostly rigid. Taken together, FEL analysis revealed the receptor’s conformational stability, with red regions indicating low-energy, stable conformations and blue regions representing high-energy, unstable states.

#### 2.4.7. Secondary Structure Analysis

**Figure 11:**
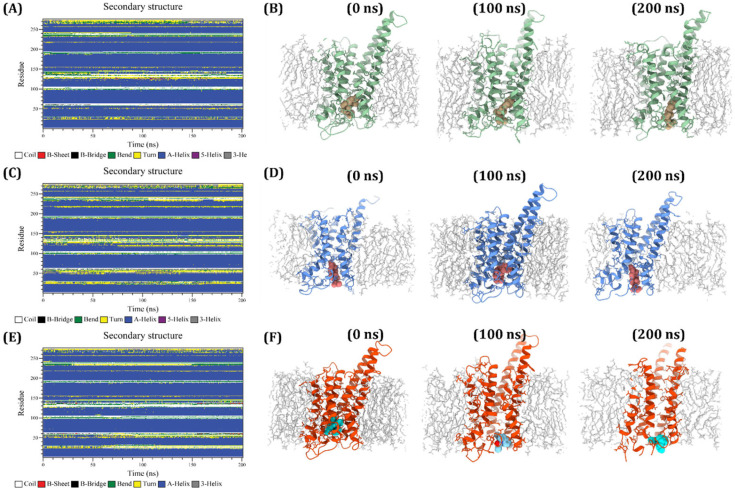
Secondary structure analysis of ligand-docked protein complexes. Time evolution of secondary structure patterns in TGR5–ergosterol (**A**), TGR5–stigmasterol (**C**), and TGR5-R399 (**E**). Structural snapshots illustrating secondary structure changes at 100 ns and 200 ns for TGR5–ergosterol (**B**), TGR5–stigmasterol (**D**), and TGR5-R399 (**F**).

The secondary structure of the docked protein complexes during simulations was evaluated using the Define Secondary Structure of Protein (DSSP) command-line tool to primarily analyse secondary-structure variations upon binding to the respective ligands. Herein, the changes of α-helix (blue) and β-sheets (red) for TGR5-APO, TGR5–ergosterol, TGR5–stigmasterol, and TGR5-R399 that occurred at the crucial time period were identified and are presented in Figure 11. In our experiment, a prominent blue colour in the TGR5-APO system indicated that the α-helix existed and would not have been affected over any period of time. Likewise, a continuous red colour would indicate that the β-sheets were also well preserved, except for the coil, which tended to become disordered (white) at ~120–150 ns. A more stable and continuous α-helix, denoted in blue colour, was seen in the TGR5–ergosterol system. The rare occurrence of a white coil would indicate that the protein was highly rigid and intact for 200 ns, with a slight, insignificant unfolding event. In contrast, a higher proportion of green and yellow colours, and often intermittent breaks in the blue colour, were observed in the TGR5–stigmasterol system. This would indicate that stigmasterol binding had induced partial local unfolding, particularly of the α-helices, which, in turn, led to the formation of additional bends and turns. This result demonstrated that protein remained in a highly flexible state rather than in a tightly ligand-bound complex. Similar to the TGR5–ergosterol system, a stable, well-defined secondary structure was maintained, as indicated by the constant thick blue bands. Minimal unfolding of helices (white colour) was observed in this system, which proved that the ligand binding was stable and did not distort the structural conformation. Further changes in the α-helices, β-sheets, coils, turns, and bends were calculated and are presented in Table S1. As demonstrated in Table S1 and Figure 11, TGR5–ergosterol showed the highest α-helical content, with very few bends and turns, indicating the highest structural stability and integrity. Comparatively, a significant decrease in the α-helical content, associated with a marked increase in turn and bend regions, was observed in the TGR5–ergosterol complex, suggesting that the ligand might induce partial destabilisation and, in turn, lead to greater flexibility. A stable secondary native structure was also maintained in the R399 complex.

#### 2.4.8. Binding Free Energy Analysis

The top-ranked complexes (TGR5–ergosterol, TGR5–stigmasterol, and TGR5-R399) binding free energies were determined using the g_mmpbsa tool. Using the last 100 ns of trajectories, the binding free energy for each system was calculated and decomposed into electrostatic, Van der Waals, polar solvation, and SASA contributions (kJ/mol). The values for these are depicted in Table 4. The evaluated binding free energy values for the TGR5–ergosterol, TGR5–stigmasterol, and TGR5-R399 complexes were −139.868 ± 12.318, −112.232 ± 13.772, and −93.424 ± 8.319, respectively. Similarly, higher negative values for the Van der Waals energy were observed for the ergosterol system (−2.04.110 ± 12.858), followed by TGR5–stigmasterol (−177.897 ± −15.250) and TGR5-R399 (−140.146 ± 10.017 kJ/mol). Generally, a more negative value would indicate a stronger interaction and greater affinity between the ligand and the protein. The calculated results suggest that ergosterol exhibited a higher binding free energy, comparable to the molecular docking results, which was particularly true of the docking score (kcal/mol). The polar solvation energy for TGR5–ergosterol was 104.124 ± 12.738 kJ/mol, while for TGR5–stigmasterol, it was 114.346 ± 16.520 kJ/mol. With the observation of lower polar solvation energy for ergosterol when compared with stigmasterol, it also favoured a suggestion that the ergosterol docked complex was comparatively much more stable than stigmasterol. Furthermore, the non-polar solvation (SASA) for ergosterol (−22.085 ± 1.213 kJ/mol) and stigmasterol (−22.435 ± 1.178 kJ/mol) were similar, indicating strong stabilisation by hydrophobic interactions. Taken together, based on hydrophobic, van der Waals, and binding energy values, ergosterol exhibited a stronger ability to bind with TGR5.

## 3. Discussion

**Figure 12:**
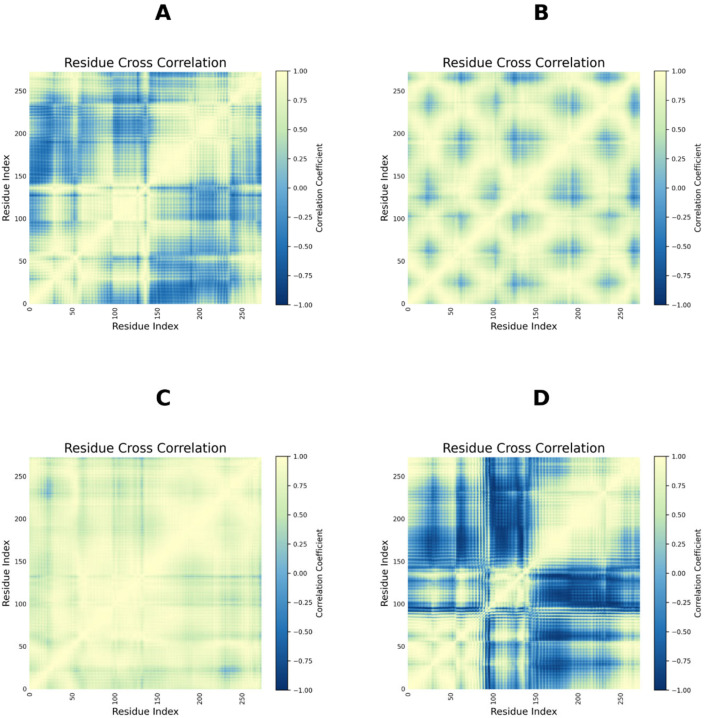
Dynamic cross-correlation matrix plot shown for (**A**) TGR5-Apo, (**B**) TGR5–ergosterol, (**C**) TGR5–stigmasterol, and(**D**) TGR5-R399.

The experimental determination and illustration of the potential activity of lead compounds against any disease can be intricate and time-consuming processes [27]. Nevertheless, this can be made a bit simpler with the use of computer-aided drug design (CADD) [27]. Herein, we employed an in-silico approach, including molecular docking and MDS, primarily to elucidate the probable mechanism underlying the anti-inflammatory potential of certain compounds present in Triphala. Molecular docking provides information on a compound’s orientation within the active site of a target protein. Furthermore, to confirm the stability of the docked complexes, MDS would be essential. In accordance with the initial docking analysis, we found that ergosterol and stigmasterol had lower negative ΔGbind values. The top two ranked compounds were subjected to MDS analysis, which enabled us to understand how ligand binding affects TGR5 stability, compactness, and amino acid behaviour, among other effects. To correlate the local flexibility of residues with dynamic coupling, the Dynamic Cross-Correlation Matrix (DCCM) was analysed for TGR5–ergosterol, as well as for stigmasterol, R399-bound, and Apo forms. As shown in Figure 12, the low flexible residues were found to be engaged in highly cooperative motions. Highly predominant positive correlations were observed across most residue pairs, indicating coordinated motions and structural stabilisation. In contrast, TGR5–stigmasterol and TGR5-R399 exhibited transitional and, to some extent, destabilising dynamic behaviour, suggesting ligand-induced conformational changes in the protein.

Inter-hydrogen bond formation is significant, as it contributes to stability and thereby lowers the complex’s binding energy. As observed, TGR5–ergosterol maintained 5 hydrogen bonds over the 200 ns simulation, with an average of 2–3 hydrogen bonds formed, affirming their interaction, binding affinity, and stability with ergosterol. To examine the specific active-site residue interaction that forms a hydrogen bond, a heatmap was generated across different simulation frames. As shown in Figure S4, Y240, T243, S270, and G269 were the crucial amino acids that formed hydrogen bond interactions. Among these, the slim and discontinuous vertical lines of Y240, G269, and S270 indicated that the formed hydrogen bonds with these amino acids were relatively transient. In comparison, T243 exhibited a highly stable hydrogen bond, as indicated by continuous blue lines. Likewise, a similar comparison was performed with TGR5–stigmasterol, in which we found that only Y240 formed a stable hydrogen bond, whereas S270, E169, and N93 rarely did so. The heatmap clearly shows that the ergosterol-binding interaction was more stable, as indicated by the greater number and frequency of amino acids that formed hydrogen bonds with TGR5. This result agrees with the more negative ΔGbind values, indicating greater stability and affinity.

**Figure 13:**
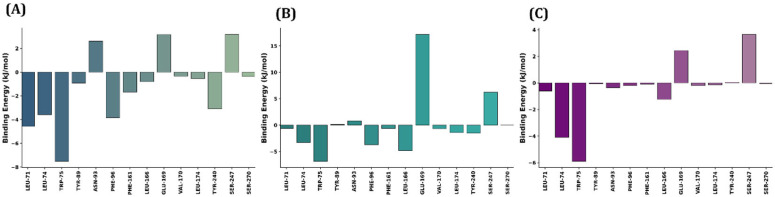
Residue-wise binding energy contribution analysis. The above bar graphs represent the per-residue binding energy contributions (kJ/mol) of the key amino acid residues involved in (**A**) TGR5–ergosterol, (**B**) TGR5–stigmasterol, and (**C**) TGR5-R399.

To further examine the role of key amino acids and the amounts of energy they contribute to ligand binding, residue-wise binding energy values were calculated for all systems. Significant active site residues, such as L71, L74, W75, Y89, N93, F96, F161, L166, E169, V170, L174, Y240, S247, and S270, which could play a crucial role in ligand binding, were selected. As has been depicted in Figure 13, based on the binding energy, the number of residues that could stabilise ligand binding was observed in the ergosterol system. For instance, W75, with ~−7.5 kJ/mol, appeared to be the strongest contributor, followed by L71, F96, and Y240 (respectively, ~−3 kJ/mol). Accordingly, L71 and F96 could have favoured hydrophobic stabilisation. Among the 14 residues, N93 and E69 exhibited positive values, suggesting the electrostatic or solvation energies upon ligand binding. Unlike the TGR5–ergosterol system, overall binding to individual residues was much weaker in the TGR5–stigmasterol system. This was because although W75, L166, and F96 possibly appeared to favour ligand binding, the positive binding energy values of S247 (~+6 kJ/mol) and E169 (~+17 kJ/mol) contributed highly unfavourably, which might have led to possible charge repulsion and loss of vital hydrogen bonds with the stigmasterol when compared with TGR5–stigmasterol. Herein, the TGR5-R399 complex yielded better results, as W75 (~−6 kJ/mol) and L74 (~−4 kJ/mol) exhibited moderate negative binding energies but remained weaker than the ergosterol-bound ones. Once again, this may have been due to the influence of E169 and Y47, whose values corresponded to approximately +2.5 and +3.7 kJ/mol, respectively.

Further structural snapshots of TGR5–ergosterol, TGR5–stigmasterol, and TGR5-R399 were taken at 0, 100, and 200 ns, and then analysed. As shown in Figure S3, ergosterol binding to the TGR5 active site did not alter the orientation of TM6. In TGR5–stigmasterol, the ligand moved sideways and upward. As a result of ligand movement, a slight partial shift in the TM6, an essential binding domain for the agonist, was noted. Likewise, a substantial change in the R399-binding site of TM6 was observed. This structural snapshot clearly showed that ergosterol binding to TGR5 did not have a pronounced effect on TM6-helix movement, thereby confirming the stabilisation of the active conformation, which remained crucial for ergosterol to elicit agonist activity. As shown in Figure 11 and Figure S3, the observed positional and orientational adjustments of ergosterol during the simulations were minimal and unlikely to affect its stability in binding to TGR5. To further affirm its stable binding, we also analysed the interaction of ergosterol with TGR5 at different time intervals during MDS, with special emphasis placed on hydrogen bond interaction. As shown in Figure S5, we found that ergosterol appeared to be present within the active site throughout the simulation. Tyr240, a crucial amino acid in TGR5, was found to form hydrogen bonds with ergosterol at 50, 100, 150, and 200 ns. Furthermore, H-bond occupancy was also calculated and has been depicted in Figure S4. The heatmap illustrates the hydrogen bonding interaction profile between ergosterol and the key binding-site residues of TGR5 during the molecular dynamic trajectory. Among the analysed residues, TYR240 exhibited the most consistent interaction, maintaining hydrogen bonding with ergosterol across a maximum portion of the simulation frames, indicating that this residue played a key stabilizing role in maintaining the ligand within the binding pocket. In contrast, ASN93, GLU169, and SER270 exhibited minimal hydrogen-bond formation, suggesting that these residues contribute to transient or auxiliary interactions rather than continuous stabilization. Overall, this heatmap would indicate that while the ligand undergoes some conformational adjustment within the receptor cavity, stable anchoring interactions—particularly with TYR240—were preserved throughout the simulation, supporting the receptor–ligand complex’s structural stability. It is also noteworthy to mention that the computed RMSD values for the initial frame (0 ns) and at 200 ns were ~0.2 nm for TGR5–ergosterol, indicating that ergosterol binding did not significantly alter the protein structure or its conformational dynamics (Table S2).

Furthermore, the structural attribute of ergosterol presumably defined its agonistic behaviour. The tetracycline backbone may facilitate hydrophobic packing within the transmembrane binding cavity, and the hydroxyl moiety present in the C3 position may cater to hydrogen bonding. In comparison, subtle structural variations in stigmasterol, specifically in the side-chain geometry, might induce steric perturbations that partially displace TM6. These findings suggest that the hydrophobic core and polar functionality of ergosterol are the key attributes for the stabilisation of the putative TGR5 agonists. Although these results provide mechanistic insights, numerous limitations of this computational study must be acknowledged. Docking scores provide only approximate binding affinity and may not provide clear entropic or electrostatic contributions. It is also noteworthy to mention that 200 ns simulations tended to reveal only short-to-mid-time dynamics, which were not sufficient to capture GPCR activation, as conformational transitions might occur beyond 200 ns. The membrane environment used in the computational study was simple and substantially simpler than the complexity of the real cellular membrane. Given these shortcomings, experimental validation is of utmost importance to justify these computational findings. For instance, in vitro TGR5 activation assays should be performed to confirm ergosterol’s agonistic activity. Additionally, to confirm the functional contribution of the key amino acids, such as W75, Y240, T243, L71, and F96, site-directed mutagenesis should be applied. Furthermore, to measure the binding affinity of ergosterol with TGR5, isothermal titration calorimetry and surface plasmon resonance could be a great option. To confirm ligand-induced TM6 stabilisation, structural studies, such as fluorescence-based conformational analysis or cryo-electron microscopy, would be mandatory. Finally, to authorise the pharmacological basis of ergosterol, cell-targeted in vivo anti-inflammatory model studies should be performed to confirm ergosterol’s agonistic activity at TGR5.

## 4. Materials and Methods

### 4.1. Ligand Preparation

The compounds present in *Terminalia chebula*, *Terminalia bellirica*, and *Phyllanthus emblica* were first confirmed in the Indian Medicinal Plants, Phytochemistry, and Therapeutics (IMPAAT) database, India (https://cb.imsc.res.in/imppat/ accessed on 9 August 2025). The 3D structures of the compounds β-sitosterol, lanosterol, dammarane, oleanane, lupane, ursane, and squalene, which have a similar backbone to sterol-like compounds, were retrieved from the PubChem Database and downloaded as 3D SDF files (https://pubchem.ncbi.nlm.nih.gov/ accessed on 9 August 2025). Before docking, all compounds’ structures were loaded into OpenBabel, their protonation states were assigned at physiological pH 7.4, and the force field “MMFF94” was used for energy minimisation. Further, they were converted into Pdb files and subsequently used for docking studies.

### 4.2. Protein Preparation

The crystal structure of the human TGR5 complexed with the ligand R399 (PDB ID: 7XTQ) was retrieved from the RCSB Protein Data Bank, and the protein preparation was performed in Biovia Discovery Studio [28]. This allowed us to process the protein suitable for further docking procedures. First, the co-crystallised compounds R399 were removed manually, followed by the removal of water molecules and other heteroatoms. Next, hydrogen atoms were added, assigning bond orders and filling in missing atoms and residues where present. Furthermore, the protonation states of His, Asp, and Glu residues were determined to optimise the hydrogen bond network.

### 4.3. Molecular Docking Analysis

First, the 3D structures of the ligands were obtained from PubChem and prepared for docking using Open Babel-2.4.1. Molecular docking was performed using AutoDock 4.2 using the Lamarckian Genetic Algorithm (LGA). The docking grid was centred on the co-crystallised ligand binding site, with grid box dimensions set to encompass the entire active cavity (7XTQ: X-96.775519 Y-121.406815 Z-114.750556) (7CXN: X-96.722937 Y-122.437469 Z-115.639844). The following parameters were used: population size of 150, maximum number of energy evaluations of 2,500,000, mutation rate of 0.02, crossover rate of 0.8, and 01s independent docking runs per ligand.

To validate the docking protocol, the co-crystallized ligands R399 (7XTQ) and INT-777 (7CXN) were redocked into their respective binding sites. The reproduced docking poses were compared with their crystallographic conformations by visual inspection of interactions and by RMSD calculations. The re-docked R399 and INT-777 showed comparable binding orientations and conserved key interactions within the active site, confirming the reliability of the docking procedure. Protein–ligand interactions were studied using 2D and 3D visualization tools to identify hydrogen bonds, hydrophobic contacts, π–alkyl, π–σ, and other non-covalent interactions. A comparative analysis of the 7XTQ and 7CXN structures was performed to assess the conservation of key active-site residues, including L71, W75, F96, L166, and Y240 [26].

### 4.4. Drug-Likeness and Prediction of Pharmacokinetics and Toxicity

The physicochemical properties of ergosterol (PubChem ID: 444679) and stigmasterol (PubChem ID: 5280794) were estimated using pkCSM (https://biosig.lab.uq.edu.au/pkcsm/ accessed on 23 August 2025). The drug-likeness parameters, including molecular weight, number of hydrogen bond acceptors and donors, rotatable bonds, solubility, and topological polar surface area (TPSA), were predicted and validated in accordance with the Lipinski rule of five. The canonical simplified molecular-input line-entry system (SMILES) for the above-mentioned compounds was used as an input for this analysis. Furthermore, the pharmacokinetic properties, such as adsorption, metabolism, excretion, and toxicity, were calculated using the online web server pkCSM (https://biosig.lab.uq.edu.au/pkcsm/ accessed on 23 August 2025). This tool provided predictions for key parameters, including gastrointestinal absorption, cytochrome P450-mediated absorption and inhibition, membrane permeability, mutagenicity, and organ toxicity (https://tox.charite.de/ accessed on 29 August 2025).

### 4.5. Molecular Dynamics

Molecular dynamics (MD) simulation, a powerful computational method that uses Newton’s laws of motion, was employed to evaluate the stability of protein–ligand docked complexes. The highest docking scores were used to select TGR5-R399, TGR5–ergosterol, TGR5–stigmasterol, and the apo protein, which were then subjected to 200 ns simulations. The simulation was performed using the GROMACS software package (2019.4) coupled with the CHARMM36 force field [29,30]. Initially, the protein–ligand minimisation step was performed in vacuum using the steepest descent algorithm. Once the system’s potential energy was minimised, the Simple Point Charge (SPC) water model was added, using a cubic periodic box to solvate the complex. Further neutralisation was achieved using the gmxgenion tool, followed by energy minimisation to optimise the structure and remove steric clashes. The complex systems were subsequently maintained with an appropriate salt concentration of 0.15 M by adding suitable numbers of Na^+^ and Cl^−^ counter ions. Once energy minimisation was complete, the system was equilibrated in two steps. In the second step of the NPT ensemble, the system density and pressure were stabilised at 1 bar and 310 K, respectively. The equilibrated phase was selected for the final 200 ns production run. Finally, the trajectory of the simulation was analysed using various tools provided by the Gromacs software package, including protein root mean square deviation (RMSD), root mean square fluctuation (RMSF), radius of gyration (RG), solvent-accessible surface area (SASA), and hydrogen bonding (H-Bond). The molecular mechanics Poisson–Boltzmann surface area (MM-PBSA) approach was employed to evaluate the binding free energy values of TGR5–ergosterol, TGR5–stigmasterol, and TGR5-R399 with the respective ligands over simulation time. A GROMACS utility, g_mmpbsa, was employed to estimate the binding free energy [25]. To obtain an accurate result, we computed TGR5-ERG and TGR5-R399 over the last 50 ns with a time step of 1000 frames. This program used a Python script, MmPbSaStat.py, to predict the average energy for each system. The ΔG was further calculated as per the following equation:

ΔG_binding_ = G_complex_ − (G_protein_ + G_ligand_) (1)

G_complex_ represents the free energy of the protein–ligand complex (PL denotes the used ligand), while G_protein_ and G_ligand_ denote the protein and ligand-free energies.

The estimation of free energy in bound and unbound forms was established by the following:

G_x_ = (E_MM_) − TS + (G_solv_) (2)

Herein, X corresponds to the PL complex or free form, P or L. EMM determines the average molecular mechanics. TS refers to the entropic contribution, and G_solv_ denotes the free energy solvation of the protein-bound with the ligand.

The molecular mechanics (EMM) were calculated by considering the electrostatic and van der Waals interactions between the protein–ligand complex, as shown in Equation (3). Therein, G_solv_ denotes the linear Poisson–Boltzmann equation for individual states (G_polar_), and solvent-accessible surface area was estimated using non-hydrophobic interactions.

E_MM_ = E_bonded_ + E_non–bonded_ (3)

G_solv_ = G_nonpolar_ + G_polar_ (4)

To quantify the energy contributions of the desired active-site-interacting amino acids in the putative agonists, a per-residue decomposition analysis was performed. To evaluate the individual residues that lie in 4 Å of the TGR5 active site contribution in terms of the free energy of binding, snapshots at a final frame of 200 ns were selected. Accordingly, L71, L74, W75, Y89, N93, F96, F161, L166, E169, V170, L174, Y240, S247, and S270 were determined to be the active site amino acids that played a crucial role in forming interactions with the putative agonists. Hence, these amino acids were considered, and the g_mmpbsa tool was employed to predict the binding energies from the MM-PBSA run. Following this, the “MmPbsaDecomp.py” script was used to determine the overall binding energy contributed by each targeted individual amino acid.

## 5. Conclusions

In conclusion, this study provides general computational evidence supporting the anti-inflammatory potential of selected phytocompounds from Triphala, with particular mention of ergosterol as a putative TGR5 agonist. Molecular docking investigations showed ergosterol and stigmasterol as top-ranking compounds, with ergosterol showing the highest binding affinity (−12.34 ± 0.17 kcal/mol), and favourable interactions with the TGR5 active site. Subsequent MDS analysis further supported the structural stability of TGR5 upon ergosterol binding, as was assessed by RMSD, RMSF, Rg, and hydrogen bond profiling. Binding free energy calculations assisted the findings, indicating the thermodynamic favourability of the interactions. In addition, PCA and FEL analyses showed that protein movement was associated with stable confirmation upon ergosterol binding, suggesting that the putative agonist did not substantially affect TGR5 dynamics. Also, the in silico ADMET predictions suggested that the selected phytocompounds, particularly ergosterol, possessed physicochemical properties consistent with drug-likeness and acceptable pharmacokinetic profiles. Although these assessments are predictive, they further support the feasibility of ergosterol as a potential therapeutic candidate. Taken together, this study demonstrates computer-assisted natural product drug discovery strategies for identifying ergosterol as a putative lead compound for TGR5 agonistic activity in anti-inflammatory drug development. Nevertheless, experimental validation through receptor activation, biochemical binding studies, and in vivo anti-inflammatory evaluations would be mandatory to fully establish this ergosterol-agonistic activity and to confirm its translational scope.

## Figures and Tables

**Table 1:** A summary of the docking scores and their interactions for the selected compounds.

Ligand	PubChem ID	Docking Score (kcal/mol)	No. of H-Bonds	H-Bond Residues	No. of Other Non-Covalent Interactions	Other Interacting Residues
R399 (control)	46195848	−9.765 ± 0.005	2	Y240, S247	11	V248, V170, L244, L174, L166, F96, N93, F92, L71, L74, F161
Ergosterol	444679	−12.34 ± 0.17	1	Y240	9	L244, L266, F66, L71, P92, W75, Y89, L166, Y251
Stigmasterol	5280794	−10.35 ± 0.04	1	S270	9	Y240, F96, T243, L266, L71, W75, Y251, F161, L166
Cycloartenol	92110	−9.725 ± 0.515	1	S157	11	L244, F96, T243, L266, L71, L74, W75, Y89, L166, Y251, F161
β-Sitosterol	222284	−9.27 ± 0.21	1	F161	7	Y240, T243, L266, F96, L71, L74, L166
Lanosterol	246983	−9.155 ± 0.025	0	None	11	F161, Y251, L166, Y89, L74, L71, W75, L266, L244, T243, Y240
Dammarane	9548714	−8.825 ± 1.355	0	None	12	F96, L244, T234, L266, Y251, L166, N93, P92, L74, W75, Y89, F161
Oleanane	9548717	−8.81 ± 0.7	0	None	10	T243, L266, L244, Y240, F96, L74, N93, Y89, F161, Y251
Lupane	9548715	−6.35 ± 0.23	0	None	11	L244, T243, Y240, L266, F96, L71, L74, Y89, F161, Y251, L166
Ursane	9548870	−5.965 ± 0.345	0	None	9	L266, T243, L244, Y240, F96, N93, Y251, L166, F161
Squalene	638072	−5.55 ± 0.555	0	None	11	V170, L166, Y251, V246, F161, L74, N93, P92, L266, L71, F96

**Table 2:** Predicted drug-likeness properties of ergosterol and stigmasterol.

Ligand (PubChem ID)	cLog P	MW (g/mol)	TPSA	nHA	nHD	nROT	Drug Likeness	Lipinski Violation
R399 (46195848)	4.372	399.3	42.43	4.0	0.0	4.0	−5.93	No
Ergosterol (444679)	5.44	396.6	20.23	1.0	1.0	4.0	−6.72	No
Stigmasterol (5280794)	6.57	412.7	20.23	1.0	1.0	5.0	−7.46	No

**Table 3:** Predicted ADMET properties of ergosterol and stigmasterol.

Ligand Name	Ligand Name	Ergosterol		Stigmasterol	
Pharmacokinetics Absorption	Caco2 permeability	16.5 × 10^−6^ cm/s	Good intestinal absorption	16.3 × 10^−6^ cm/s	High permeability
	Intestinal absorption (human)	High	Excellent absorption	High	Excellent absorption
	Skin permeability	1.55 × 10^−6^ cm/s	Easily permeates skin	1.65 × 10^−3^ cm/s	Easily permeates skin
	P-glycoprotein substrate	-	No	-	No
	P-glycoprotein I inhibitor	-	Yes	-	Yes
	P-glycoprotein II inhibitor	-	Yes	-	Yes
Distribution	BBB permeability	5.82	High brain penetration	5.89	High brain penetration
	CNS permeability	0.0177	Low–moderate CNS permeability	0.0223	Low–moderate CNS permeability
Metabolism	CYP2D6 substrate	-	No	-	No
	CYP3A4 substrate	-	Yes	-	Yes
	CYP1A2 inhibitor	-	No	-	No
	CYP2C19 inhibitor	-	No	-	No
	CYP2C9 inhibitor	-	No	-	No
	CYP2D6 inhibitor	-	No	-	No
	CYP3A4 inhibitor	-	No	-	No
Excretion	Total Clearance	3.66 mL/min/kg	Moderate clearance	4.15 mL/min/kg	Moderate clearance
	Renal OCT2 substrate	-	No	-	No
Toxicity	AMES toxicity	-	No	-	No
	Max. tolerated dose (human)	0.2037 mg/kg/day	Low MTD	0.217 mg/kg/day	Low MTD
	hERG I inhibitor	-	No	-	No
	hERG II inhibitor	-	Yes	-	Yes
	Oral rat acute toxicity (LD_50_)	179.4 mol/kg	Indicates very low acute toxicity	347 mol/kg	Indicates very low acute toxicity
	Oral rat chronic toxicity (LOAEL)	7.63 mg/kg/day	Moderate chronic toxicity level	7.45 mg/kg/day	Moderate chronic toxicity level
	Hepatotoxicity	-	No	-	No
	Skin sensitisation	-	No	-	No
	Mutagenicity	-	Inactive	-	Inactive
	Cytotoxicity	-	Inactive	-	Inactive
Nuclear receptor signalling pathway	Aryl hydrogen receptor	-	Inactive	-	Inactive
	AR	-	Inactive	-	Inactive
	ARLBD	-	Inactive	-	Inactive
	Aromatase	-	Inactive	-	Inactive
	ER-α	-	Inactive	-	Inactive
	ERLBD	-	Inactive	-	Inactive
	PPAR-γ	-	Inactive	-	Inactive
Stress response pathway	nrf2/ARE	-	Inactive	-	Inactive
	HSEs	-	Inactive	-	Inactive
	Mitochondrial membrane potential	-	Active	-	Inactive
	p53	-	Inactive	-	Inactive
	ATAD5	-	Inactive	-	Inactive

**Table 4:** MMPBSA predicted values for the protein–ligand complexes. The determined energy values are presented as shown.

System	Van der Waals Energy (kJ/mol)	Electrostatic Energy (kJ/mol)	Polar Solvation Energy (kJ/mol)	SASA Energy (kJ/mol)	Binding Energy (kJ/mol)
TGR5–ergosterol	−204.11 ± 12.86	−17.80 ± 3.23	104.12 ± 12.74	−22.08 ± 1.21	−139.87 ±12.32
TGR5–stigmasterol	−177.90 ± 15.25	−23.25 ± 5.25	111.35 ± 16.52	−22.44 ± 1.18	−112.23 ± 13.77
TGR5-R399	−140.15 ± 10.02	−20.96 ± 5.22	83.810± 12.13	−16.12 ± 1.03	−93.42 ± 8.92

## Data Availability

The data presented in this study are available from the corresponding author upon request.

## Supplementary Materials

The following supporting information can be downloaded at: https://www.mdpi.com/article/10.3390/ijms27073130/s1.
