# Supplementary material for: Computational Identification of Triphala-Derived Sterol Compounds as Putative Agonists of the Human Takeda G Protein-Coupled Receptor (TGR5)
Source: Int J Mol Sci. 2026 Mar 30;27(7):3130. doi: 10.3390/ijms27073130 (PMC13073596; doi:10.3390/ijms27073130)
Supplement: Supplementary file 1 [file ijms-27-03130-s001.zip › ijms-4163665-supplementary.pdf]

Supplementary Materials

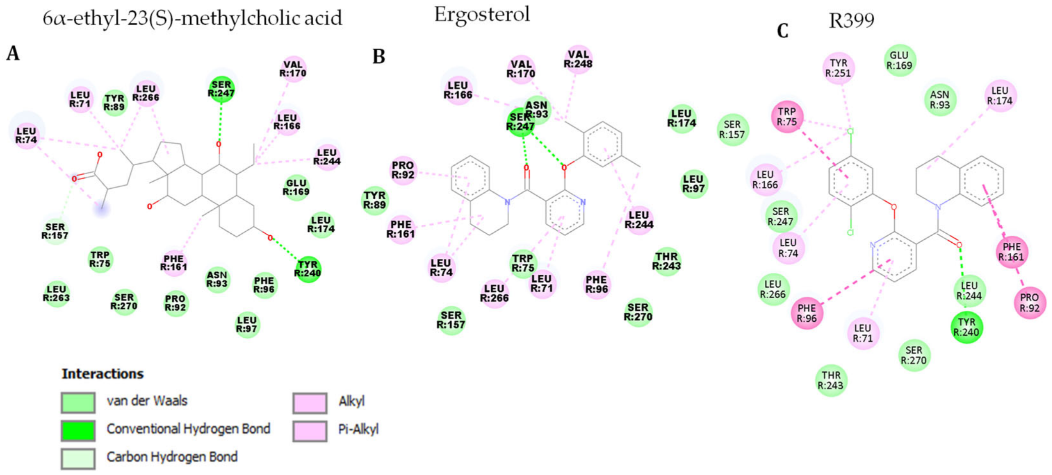

Figure S1. 2D ligand–protein interaction diagrams of compounds bound to the receptor binding pocket. **(A)** Interaction profile of 6α-ethyl-23(S)-methylcholic acid within the binding site showing key contacts with surrounding amino acid residues. **(B)** Interaction map of ergosterol with receptor active site residues. **(C)** Binding interactions of R399 within the same binding pocket. In all panels, the ligands are represented in stick form at the center, while interacting amino acid residues are shown as labeled circles around the ligand. Green dashed lines represent conventional hydrogen bonds, light green shading indicates van der Waals interactions, and pale green lines denote carbon–hydrogen bonds. Pink dashed lines represent hydrophobic interactions, including alkyl and π–alkyl contacts with residues such as leucine, phenylalanine, and tryptophan. These interactions collectively stabilize ligand binding within the receptor pocket and highlight key residues contributing to ligand recognition and affinity.

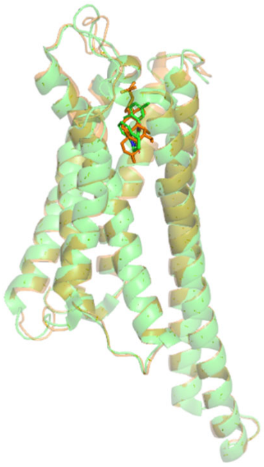

Figure S2. Structural superimposition of the native and redocked ligand within the receptor binding pocket used for docking validation. The crystal structure of the receptor (PDB ID: 7XTQ) is shown in green, while the redocked conformation is displayed in yellow. The redocked ligand is represented as orange sticks within the binding cavity. The close overlap between the crystallographic pose and the redocked pose indicates that the docking protocol reliably reproduces the experimentally observed binding orientation, supporting the validity of the molecular docking procedure

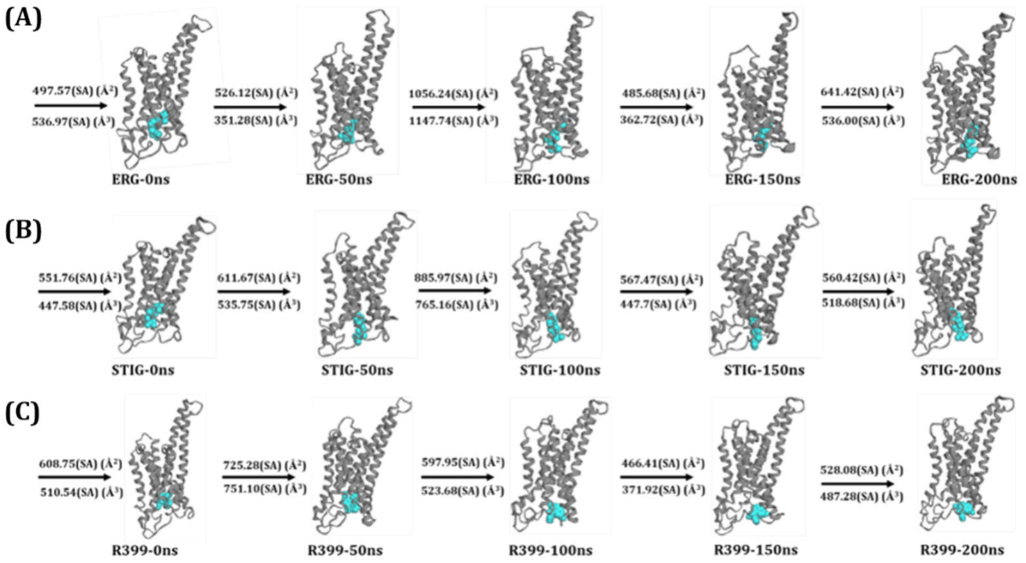

Figure S3. Time-dependent structural snapshots of the protein–ligand complex during molecular dynamics simulation. Representative conformational snapshots of **(A)** TGR5-Ergosterol, **(B)** TGR5stigmastrol, and **(C)** TGR5-R399 complex captured at different time intervals (0ns,50 ns, 100 ns, 150 ns, and 200 ns).

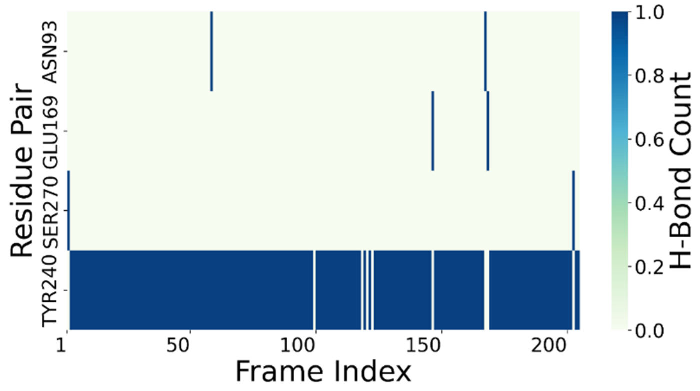

Figure S4. Hydrogen-bond interaction heatmap of the TGR5–ergosterol complex during molecular dynamics simulation. The x-axis corresponds to the simulation frame index, while the y-axis lists the interacting amino-acid residues (ASN93, GLU169, SER270, and TYR240). The colour scale indicates the presence of hydrogen bonds, with darker blue regions representing frames in which hydrogen bonding occurred.

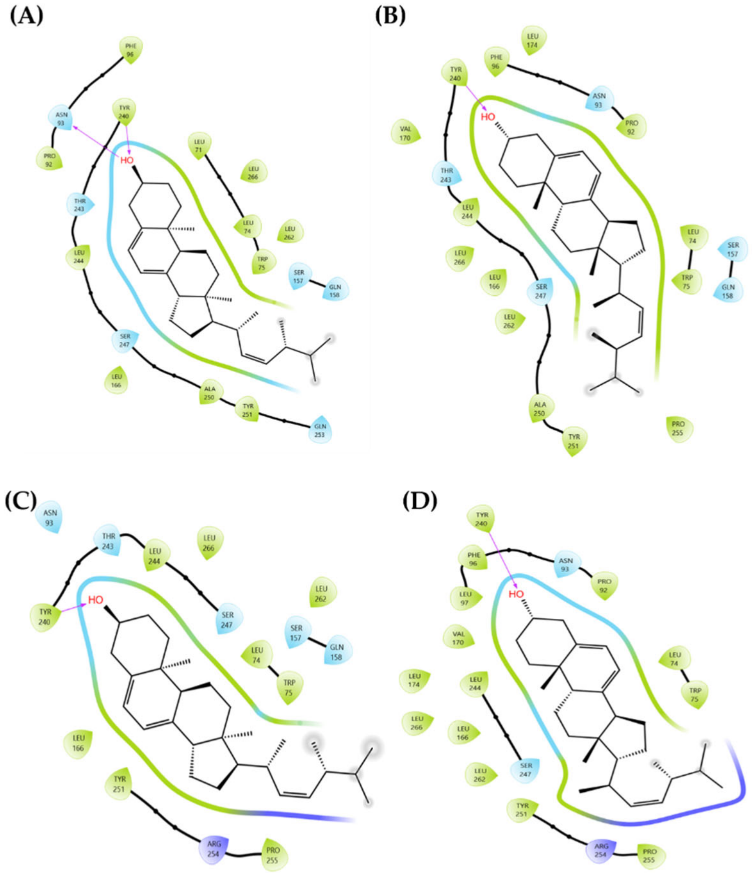

Figure S5. Two-dimensional ligand–receptor interaction diagrams of ergosterol bound within the binding pocket of TGR5 at different time intervals of MDS. Panel **(A)** illustrates the 2D interaction profiles of the ergosterol bound with TGR5 at 50 ns. Panel **(B)** illustrates the 2D interaction profiles of the ergosterol bound with TGR5 at 100 ns, Panel **(C)** illustrates the 2D interaction profiles of the ergosterol bound with TGR5 at 150 ns, and Panel **(D)** illustrates the 2D interaction profiles of the ergosterol bound with TGR5 at 200 ns. Hydrogen-bond interactions are represented by red arrows.

Table S1. The calculated percentage of secondary structure values from the MDS analysis of TGR5-Apo, TGR5-Ergosteol, and TGR5-Stigmasterol docked complexes.

System	α-Helix (%)	β-Sheet (%)	Coil (%)	Turn (%)	Bend (%)
**TGR5-Apo**	45.26	6.04	44.81	3.80	0.09
**TGR5-Ergosterol**	45.38	5.57	45.36	3.62	0.07
**TGR5-Stigmasterol**	40.41	5.77	44.51	5.72	3.60
**TGR5-R399**	45.21	6.31	44.53	3.83	0.13

Table S2. Average RMSD (å) of protein backbone atoms over different simulation time intervals (0–50, 0–100, 0–150, and 0–200 ns) for ergosterol, stigmasterol, and R399-bound complexes, indicating their structural stability during molecular dynamics simulations.

RMSD (Å)			
**Time (ns)**	**Ergosterol**	**Stigmasterol**	**R399**
0-50	1.156	1.945	1.444
0-100	1.403	1.632	1.880
0-150	1.458	1.635	1.958
0-200	1.351	1.620	2.225
